# Circulating microparticle levels are reduced in patients with ARDS

**DOI:** 10.1186/s13054-017-1700-7

**Published:** 2017-05-25

**Authors:** Ciara M. Shaver, Justin Woods, Jennifer K. Clune, Brandon S. Grove, Nancy E. Wickersham, J. Brennan McNeil, Gregory Shemancik, Lorraine B. Ware, Julie A. Bastarache

**Affiliations:** 1 Division of Allergy, Pulmonary, and Critical Care Medicine, Vanderbilt University Medical Center, 1161 21st Ave South, Medical Center North T-1218, Nashville, 37232 Tennessee USA; 2 Department of Pathology, Microbiology, and Immunology, Vanderbilt University Medical Center, Nashville, TN USA

**Keywords:** Acute respiratory distress syndrome, Sepsis, Microparticles, Coagulation

## Abstract

**Background:**

It is unclear how to identify which patients at risk for acute respiratory distress syndrome (ARDS) will develop this condition during critical illness. Elevated microparticle (MP) concentrations in the airspace during ARDS are associated with activation of coagulation and in vitro studies have demonstrated that MPs contribute to acute lung injury, but the significance of MPs in the circulation during ARDS has not been well studied. The goal of the present study was to test the hypothesis that elevated levels of circulating MPs could prospectively identify critically ill patients who will develop ARDS and that elevated circulating MPs are associated with poor clinical outcomes.

**Methods:**

A total of 280 patients with platelet-poor plasma samples from the prospective Validating Acute Lung Injury biomarkers for Diagnosis (VALID) cohort study were selected for this analysis. Demographics and clinical data were obtained by chart review. MP concentrations in plasma were measured at study enrollment on intensive care unit (ICU) day 2 and on ICU day 4 by MP capture assay. Activation of coagulation was measured by plasma recalcification (clot) times.

**Results:**

ARDS developed in 90 of 280 patients (32%) in the study. Elevated plasma MP concentrations were associated with reduced risk of developing ARDS (odds ratio (OR) 0.70 per 10 μM increase in MP concentration, 95% CI 0.50–0.98, *p* = 0.042), but had no significant effect on hospital mortality. MP concentration was greatest in patients with sepsis, pneumonia, or aspiration as compared with those with trauma or receiving multiple blood transfusions. MP levels did not significantly change over time. The inverse association of MP levels with ARDS development was most striking in patients with sepsis. After controlling for age, presence of sepsis, and severity of illness, higher MP concentrations were independently associated with a reduced risk of developing ARDS (OR 0.69, 95% CI 0.49–0.98, *p* = 0.038). MP concentration was associated with reduced plasma recalcification time.

**Conclusions:**

Elevated levels of circulating MPs are independently associated with a reduced risk of ARDS in critically ill patients. Whether this is due to MP effects on systemic coagulation warrants further investigation.

**Electronic supplementary material:**

The online version of this article (doi:10.1186/s13054-017-1700-7) contains supplementary material, which is available to authorized users.

## Background

Acute respiratory distress syndrome (ARDS) remains common and carries significant morbidity and mortality [1] despite lung-protective ventilator strategies [2] and conservative fluid management [3]. One potential reason for the lack of proven clinical therapies is that patients are typically well into their clinical illness (48–72 h) before being enrolled in clinical trials, which may be beyond the time frame for successful therapeutic intervention. Compounding this problem is a lack of good predictors of development of ARDS in at-risk patients. One potential solution is the identification of biomarkers associated with development of ARDS in critically ill patients so that therapies can be started earlier in the most vulnerable patients. Although several biomarker studies of ARDS have been completed, no single biomarker has been predictive of development of ARDS [4]. In this study, we examined circulating microparticles (MPs) and coagulation activation in patients at risk for ARDS to determine if these biomarkers might be useful for identifying ARDS in at-risk patients and to determine if these biomarkers are associated with poor outcomes in patients who develop ARDS.

Activation of systemic coagulation is an early feature of ARDS [5] that contributes to microvascular thrombosis and organ dysfunction. MPs are central to activation and propagation of thrombosis through exposure of negatively charged phospholipids on their surface and delivery of tissue factor (TF) to the site of thrombosis [6–10]. We previously observed that patients with ARDS had higher levels of MPs in the airspace than a critically ill control group of subjects with cardiogenic pulmonary edema and that higher levels were associated with worse clinical outcomes [11]. Further, we found that these MPs contain TF and have procoagulant activity. On the basis of these results showing an important role of MPs in coagulation and ARDS in the airspace, we hypothesized that circulating MPs would be higher in at-risk patients who develop ARDS than in those who do not develop ARDS. Further, we hypothesized that higher levels of circulating MPs would correlate with severity of ARDS, be associated with increased activation of coagulation, and predict poor clinical outcomes.

## Methods

### Study subjects

This is a substudy of the Validating Acute Lung Injury biomarkers for Diagnosis (VALID) prospective cohort study of subjects admitted to the medical, surgical, or trauma intensive care unit (ICU) who are at risk for ARDS [12, 13]. Adult critically ill patients were included in VALID if they remained in the ICU for at least 2 days and were excluded if they had a history of severe chronic lung disease or were admitted for uncomplicated overdose [12, 13]. Informed consent was obtained for sample collection from the patients or their designated surrogates; if patients or surrogates were unable to provide consent, the institutional review board granted a waiver of consent. We studied 280 patients enrolled from 2010 to 2013 for whom a platelet-poor plasma (PPP) sample was available from either the enrollment day (ICU day 2) or ICU day 4. PPP was chosen for this study to avoid post-collection release of MPs by platelets during plasma processing. Demographic and clinical data were collected at enrollment on the morning of ICU day 2 and daily for 3 days. Subjects were classified as having ARDS if they met American-European Consensus Criteria (AECC) [14] for ALI or ARDS on at least 1 day in the first 4 days of their ICU admission. In accordance with the Berlin criteria for ARDS, in this article, the ARDS group includes patients with AECC ALI or ARDS. Subjects with sepsis were defined using Sepsis-2 criteria [15].

### Sample collection

PPP was prepared within 1 h of specimen collection from blood collected in citrate on ICU days 2 (study enrollment) and 4. Blood was centrifuged at 1500 × *g* at room temperature for 15 minutes to isolate plasma. Platelets were then removed by centrifugation of plasma at 13,000 × *g* for 2 minutes. The PPP supernatant was carefully collected and stored at −80 °C until further analysis.

### MP measurement

MP concentration was measured in duplicate in PPP by using an MP capture assay (ZYMUPHEN MP-Activity assay; Aniara Diagnostica, West Chester, OH, USA). In this assay, plasma samples are incubated in microtiter plates coated with streptavidin and biotinylated annexin V. Then, factors Xa and Va, calcium, and prothrombin are added. Finally, thrombin is detected by a chromogenic thrombin substrate. Thus, this assay quantifies MPs that express phosphatidylserine on their surface.

### Plasma procoagulant activity

Plasma procoagulant activity was measured by plasma recalcification time (clot time) [16] using a mechanical clot detection system (STart 4 coagulometer; Diagnostica Stago, Asnières-sur-Seine, France). Briefly, 50 μl of PPP was incubated for 15 minutes at 37 °C. Samples were then incubated with 50 μl of pooled normal plasma (Fisher Diagnostics, Middletown, VA, USA) for 2 minutes. Clot time was determined as recalcification time following the addition of 25 mM calcium chloride. Measurements were done in duplicate.

### Statistical analysis

MP concentrations were compared across groups using Kruskal-Wallis or Mann-Whitney *U* tests as appropriate. A *p* value less than 0.05 was considered significant. Multivariable logistic regression was used to assess independent risk factors for ARDS development.

## Results

### Characteristics of subjects who did and did not develop ARDS

Table 1 describes the study population of 280 subjects. Of this cohort, 90 patients (32%) developed ARDS during the study period. The majority of patients (85%) developed ARDS within the first 2 days in the ICU. Severe trauma was the most common risk factor for ARDS. Patients who developed ARDS (*n* = 90) were more likely to be mechanically ventilated and had lower ratios of partial pressure of oxygen in arterial blood to fraction of inspired oxygen and peripheral capillary oxygen saturation to fraction of inspired oxygen, as well as higher Acute Physiology and Chronic Health Evaluation II (APACHE II) scores, than subjects who did not develop ARDS (*n* = 190). Patients with ARDS had fewer ventilator-free days than, but similar overall mortality to, those without ARDS.

**Table 1:** Characteristics of patients at risk for developing acute respiratory distress syndrome

Variable	Subjects who do not develop ARDS (*n* = 190)	Subjects who develop ARDS (*n* = 90)	*p* Value
Age, years	54 (40–66)	54 (44–64)	0.769
Male sex	64%	60%	0.598
ARDS risk factor (*n*)			
Sepsis	44	18	
Pneumonia	14	14	
Severe trauma	81	37	
Multiple transfusions	12	3	
Aspiration	6	12	
Mechanically ventilated	66%	83%	0.003
PaO_2_/FiO_2_ ratio	222 (129–309)	141 (95–214)	<0.001
SpO_2_/FiO_2_ ratio	223 (147–256)	154 (93–235)	<0.001
APACHE II score	25 (20–31)	27 (23–33)	0.017
MPs at ICU day 2, μM	5.0 (2.1–12.1)	2.9 (1.7–7.5)	0.011
MPs at ICU day 4, μM	4.5 (2.1–9.2)	4.2 (2.1–7.2)	0.430
Clot time at ICU day 2 (sec)	212 (185–250)	218 (194–255)	0.227
Clot time at ICU day 4 (sec)	228 (192–254)	252 (221–299)	0.014
Ventilator-free days	24 (15–27)	19 (7–23)	<0.001
Hospital mortality	16%	23%	0.138

*Abbreviations: ARDS* Acute respiratory distress syndrome, *MP* Microparticle, *APACHE II* Acute Physiology and Chronic Health Evaluation II, *FiO*_*2*_ Fraction of inspired oxygen, *ICU* Intensive care unit, *PaO*_*2*_ Partial pressure of oxygen in arterial blood, *SpO*_*2*_ Peripheral capillary oxygen saturation, *IQR* Interquartile range

Data are presented as median or number

### MPs were lower in subjects who developed ARDS

**Fig. 1:**
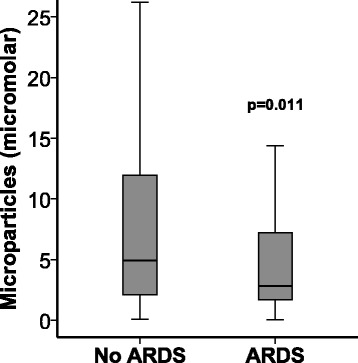
Development of acute respiratory distress syndrome (ARDS) is associated with reduced circulating microparticle concentrations. Microparticle concentrations were measured in platelet-poor plasma samples obtained at study enrollment on the morning after intensive care unit admission. Development of ARDS was evaluated daily for 4 days. *n* = 177 without ARDS, *n* = 81 with ARDS

**Fig. 2:**
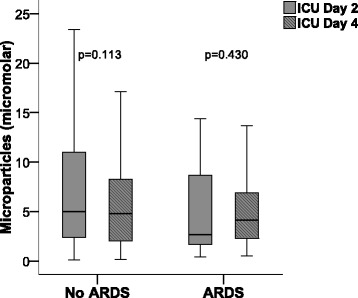
The concentration of circulating microparticles did not change during the 4-day study period. Microparticle concentrations were measured in platelet-poor plasma samples obtained at study enrollment on intensive care unit (ICU) day 2 and ICU day 4. *n* = 89

Contrary to our hypothesis, circulating MP concentrations measured at enrollment were lower in the plasma of patients who developed ARDS compared with those who did not develop ARDS (Fig. 1). The odds ratio (OR) for ARDS development was reduced by 30% per 10 μM increase in circulating MP concentration (OR 0.70 per 10 μM increase in MP concentration, 95% CI 0.50–0.98, *p* = 0.042). There was no change in MP concentration between ICU day 2 and ICU day 4 in either patients who develop ARDS or in those who did not develop ARDS (Fig. 2).

### Enrollment MP concentrations varied by risk factor for ARDS

**Fig. 3:**
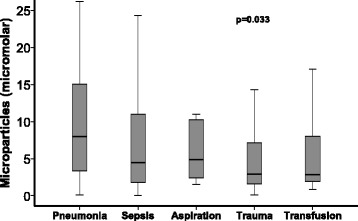
Circulating microparticle concentrations differed according to risk factors for acute respiratory distress syndrome (ARDS). Patients with pneumonia had the highest microparticle concentrations, whereas patients receiving multiple transfusions had the lowest. Microparticle concentration was measured at study enrollment. ARDS risk factors were obtained prospectively by chart review. *n* = 27 for pneumonia, *n* = 61 for sepsis, *n* = 14 for aspiration, *n* = 106 for trauma, and *n* = 13 for multiple transfusions

To test whether there were differences in circulating MP concentrations depending on risk factor for ARDS, we compared MP concentrations according to risk factors for ARDS. Subjects with pneumonia as a risk factor had the highest concentration of circulating MPs and those receiving multiple transfusions had the lowest (Fig. 3).

### MPs are inversely associated with ARDS development in subjects with sepsis

**Fig. 4:**
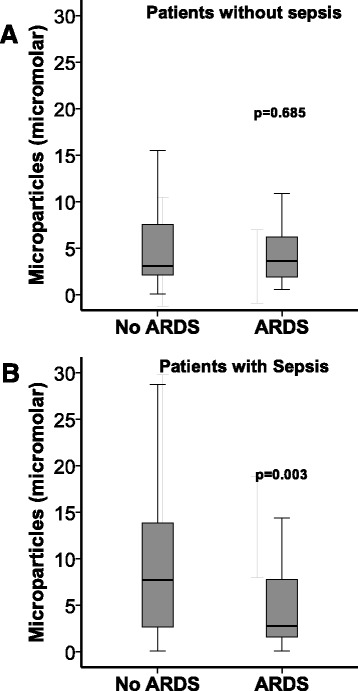
The association between lower circulating microparticle concentrations and development of acute respiratory distress syndrome (ARDS) was restricted to those patients with sepsis. **a** Microparticle concentration was not associated with ARDS in patients without sepsis. **b** Lower microparticle concentrations were associated with ARDS development in patients with sepsis. Microparticle concentrations were measured on ICU day 2, and ARDS was determined prospectively by chart review

Because of the high rate of ARDS in patients with sepsis [17, 18] and the association between sepsis and MP release [19–21], we studied the association of MPs with ARDS development in subjects with sepsis compared with those without sepsis. Lower MP concentrations were significantly associated with ARDS development in subjects with sepsis, but not in those without sepsis (Fig. 4a and b).

### Enrollment MP concentration is independently associated with ARDS development

To determine whether circulating MPs were independently associated with the development of ARDS, we used multivariable logistic regression for the development of ARDS during the study period. After controlling for age, severity of illness (APACHE II), and the presence of sepsis, a lower plasma MP level at enrollment was independently associated with development of ARDS (Table 2). A post hoc analysis shows that the relationship between MP concentration and ARDS development is preserved after controlling for ARDS risk factor (Additional file 1: Table S1).

**Table 2:** Microparticle concentration is independently associated with risk of acute respiratory distress syndrome development

Variable	OR	95% CI	*p* Value
Age (per year)	0.993	0.977–1.009	0.414
APACHE II (per point)	1.038	1.001–1.075	0.045
Sepsis	1.200	0.686–2.009	0.522
Microparticles (per 10 μM)	0.693	0.490–0.980	0.038

*APACHE II* Acute Physiology and Chronic Health Evaluation II

### Circulating MP levels correlate modestly with markers of coagulation

**Fig. 5:**
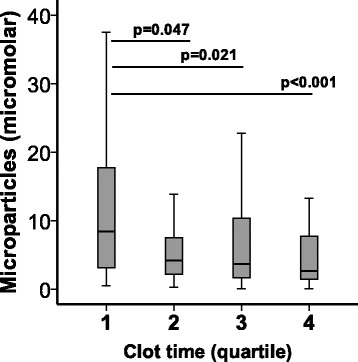
Microparticles are associated with activation of plasma coagulation. Circulating microparticle concentration decreased by quartile of clot time, with quartile 1 having the shortest clot times, representing greater activation of coagulation

Because MPs in the airspace of patients with ARDS have strong procoagulant activity, we measured whether circulating MPs were associated with plasma markers of coagulation. Patients with shorter plasma clot times, a measure of plasma procoagulant activity, had greater plasma levels of MPs than those with longer clot times (Fig. 5).

## Discussion

In this prospective study of patients at risk of developing ARDS, lower levels of circulating MPs were associated with the development of ARDS in critically ill patients at risk for ARDS. The association between lower plasma levels of MPs and the development of ARDS was independent of age, sepsis, and severity of illness. MP concentration differed by risk factor for ARDS and was stable between enrollment on ICU day 2 and ICU day 4. Interestingly, the inverse association between MP levels and development of ARDS was strongest in the subgroup of patients with sepsis.

The role of MPs in the development and evolution of ARDS is not well understood. Several in vitro studies have shown that MPs from different sources can induce inflammation and injury in lung endothelial cells. MPs from stimulated THP-1 monocytes in culture induce apoptosis in human pulmonary microvascular endothelial cells (hPMVECs) [22]. Similarly, platelet-derived MPs promote polymorphonuclear leukocyte-induced hPMVEC death [23]. In addition, MPs from apoptotic monocytes induce TF expression and platelet adhesion to human vascular endothelial cells [24]. In vivo, MPs have been shown to contribute significantly to experimental lung injury. Rats subjected to high tidal volume ventilation had increased circulating MPs after 4 h of ventilation [25]. Li et al. showed that circulating MPs isolated from the blood of lipopolysaccharide (LPS)-treated rats were sufficient to induce lung inflammation and cytokine production when injected either intravenously or intratracheally [26]. Similarly, mice and rats treated with MPs derived from cultured endothelial cells caused lung inflammation and permeability [27, 28]. On the basis of these studies demonstrating an injurious role for MPs in lung and vascular injury, we hypothesized that increased MP levels would be associated with increased risk of ARDS. However, we found instead that patients with lower circulating MPs were at increased risk of developing ARDS. This was a surprising observation and highlights the complexity of the impact of MPs on lung injury. Our findings are consistent with one previous clinical study of circulating MPs in patients with ARDS which showed that higher levels of endothelium-derived MPs were associated with reduced mortality in patients with ARDS [29]. Our data suggest that the effects of MPs may differ on the basis of specific clinical situations; for example, MPs were inversely associated with ARDS development only in patients with sepsis and not in those without sepsis. Further studies are necessary to fully understand the mechanisms that underlie these observations.

The reason for the contradiction between clinical studies of MPs in ARDS and experimental studies of MPs in culture or animal models is uncertain. One possibility is that there are subpopulations of MPs from some cellular sources that are protective. For example, MPs derived from human mesenchymal stem cells were protective against *Escherichia coli*-induced ALI when administered either intratracheally or intravenously [30, 31], whereas MPs from platelets, monocytes, or endothelial cells are injurious [22, 23, 27, 28, 32]. Second, it may be that bioactive MPs with exposed phospholipids generated in the setting of acute inflammation are sequestered in organs such as the lung rather than in the circulation. Whereas circulating MPs are lower in subjects with ARDS, MPs attached to the endothelium in the lung or other organs may be increased. Consistent with this hypothesis, MPs can be sequestered in developing thrombi through expression of adhesion molecules [33, 34]. Third, MP isolation and preparation for experimental use may alter MPs in such a way that they become proinflammatory. Supporting this concept, most studies using isolated MPs demonstrate injurious effects, whereas clinical studies of MPs show protective effects. In addition, our MP capture assay measures only MPs with phosphatidylserine on the external surface; this may be a subset of total MPs. Thus, different studies may measure different populations of MPs, depending on choice of measurement method. Fourth, in our clinical study, we compared MP levels between critically ill patients who develop ARDS and critically ill patients who do not develop ARDS, rather than comparing MP levels with those of healthy control subjects or a less ill hospitalized population. It may be that the degree of underlying illness is an important confounding variable that explains differences between human and animal studies of MPs. Finally, although we did not detect any significant changes in circulating MP concentration over 4 days in the ICU, it is possible that the timing of MP measurement is critical for evaluation of the pathogenic or prognostic roles of MPs. Measurement of MPs at the time of enrollment in the VALID study on the morning of ICU day 2 is already many hours into critical illness and often several days after initiation of symptoms. Perhaps MPs are higher in the very early stages of illness and have started to decrease by the time subjects are admitted to the ICU.

Another surprising finding was the only modest association between MP concentration and plasma procoagulant activity as measured by plasma recalcification time. Our group has previously shown that MPs in the airspace in ARDS have very high levels of TF and are highly procoagulant [11]. Thus, it was surprising that MPs were not more strongly associated with procoagulant activity in the circulation. The implication of this finding is twofold. First, it highlights an important difference between the circulation and the airspace and confirms that findings in one compartment cannot be extrapolated to another. The importance of compartmentalization of mediators has been an active and growing area of research. For example, loss of TF (the initiator of the extrinsic coagulation cascade) is protective in mice exposed to systemic LPS [35], but it was associated with worse lung injury when the LPS was delivered intratracheally [36]. Similarly, it is increasingly recognized that there are biologic and physiologic differences between ARDS with direct causes (pneumonia, aspiration) and ARDS with indirect causes (nonpulmonary sepsis, trauma) [37]. Second, the lack of association between MPs and coagulation markers suggests that it may be a noncoagulant subpopulation of MPs that regulates endothelial function and inflammation in the circulation.

Our study has some limitations. First, we measured MPs using a MP capture assay that measures the concentration of phosphatidylserine-positive MPs and does not provide information about MP cell of origin. In the study which showed that lower MP levels were associated with increased ARDS mortality, circulating MPs originated predominantly from leukocytes [29]. These MPs were characterized by flow cytometry rather than by capture assay and may have included more diverse populations of MPs. Second, although larger than samples in other clinical studies of MPs in ARDS, our sample size was limited to 90 patients with ARDS, which may limit detection of important associations of MPs and ARDS. The effects of MPs may differ depending on the overall clinical scenario. In addition, our study, although prospective, does not provide mechanistic information about the specific function of MPs. The unexpected inverse relationship between plasma MP levels and the development of ARDS will help to inform future mechanistic studies aimed at measuring MP sequestration in organs including the lung and the presence of circulating protective MPs. Whether circulating MPs affect development of organ failure outside the lung remains uncertain.

## Conclusions

In summary, lower levels of circulating MPs early after ICU admission are independently associated with the development of ARDS, and this relationship is strongest in patients with sepsis. The present findings, in conjunction with a study by Guervilly et al. [29] showing a protective association between MP concentration and mortality in ARDS, suggest that the potential role of MPs in ARDS pathogenesis is complex and not well modeled in current experimental studies. Whether MP measurements could be used clinically to aid with diagnosis or prognosis of ARDS requires further study.

## Additional file

Additional file 1: Table S1. Post hoc analysis of the effect of ARDS risk factor on the relationship between microparticle concentration and ARDS development. (DOC 33 kb)

## Abbreviations

AECC: American-European Consensus Criteria
ALI: Acute lung injury
APACHE II: Acute Physiology and Chronic Health Evaluation II
ARDS: Acute respiratory distress syndrome
FiO_2_: Fraction of inspired oxygen
hPMVEC: Human pulmonary microvascular endothelial cell
ICU: Intensive care unit
LPS: Lipopolysaccharide
MP: Microparticle
PaO_2_: Partial pressure of oxygen in arterial blood
PPP: Platelet-poor plasma
SpO_2_: Peripheral capillary oxygen saturation
TF: Tissue factor
VALID: Validating Acute Lung Injury biomarkers for Diagnosis study
