# Supplementary material for: Circulating microparticle levels are reduced in patients with ARDS
Source: Crit Care. 2017 May 25;21:120. doi: 10.1186/s13054-017-1700-7 (PMC5445431; doi:10.1186/s13054-017-1700-7)
Supplement: Additional file 1: Table S1. — Post hoc analysis of the effect of ARDS risk factor on the relationship between microparticle concentration and ARDS development. (DOC 33 kb) [file 13054_2017_1700_MOESM1_ESM.doc]

**Additional file 1:** Table S1. Post-hoc analysis of the effect of ARDS risk factor on the relationship between microparticle concentration and ARDS development.

| **Variable** | **Odds ratio** | **95% Confidence Interval** | **P value** |
| --- | --- | --- | --- |
| **Age (per year)** | 0.992 | 0.975-1.009 | 0.361 |
| **APACHE II (per point)** | 1.048 | 1.006-1.091 | 0.024 |
| **ARDS risk factor** |  |  |  |
| **Sepsis** | Referent | Referent | 0.008 |
| **Pneumonia** | 0.226 | 0.065-0.788 | 0.020 |
| **Trauma** | 0.817 | 0.204-3.268 | 0.775 |
| **Transfusions** | 0.280 | 0.085-0.925 | 0.037 |
| **Aspiration** | 0.046 | 0.005-0.476 | 0.010 |
| **Microparticles (per 10 μM)** | 0.677 | 0.463-0.991 | 0.045 |
